# Annotations

**Published:** 1908-01-04

**Authors:** 


### ANNOTATIONS.

### Certification of Inebriates.

At the last meeting of the Medico-Legal Society a discussion of much interest was introduced by a paper on "The Radical Cure: Certification of Inebriates" by Dr. Claye Shaw. The paper dealt more particularly with the problem created by persons who are admitted to asylums when suffering from an acute form of insanity as the result of alcoholic intoxication. Such persons usually rapidly recover and have to be discharged. Only too frequently they return to habits which involve danger to their own health and much suffering and misery to their relatives and friends; and perhaps, also, a return on one or more occasions to the care of an asylum. Dr. Shaw's desire is to formulate a scheme which will break this vicious circle, and will afford a chance of redemption to the victim of the drink habit. Hence he proposes that a person who has recovered from an attack of acute alcoholic insanity may, on certain conditions, be removed from the asylum, but kept under a modified form of restraint. He would, in short, permit such a patient to be detained in an inebriate home for a period of six months or longer on the certificate of two medical men. Obviously such a proposal puts considerable power and heavy responsibility on those who would be called upon to sign such certificates, and unless the law would guarantee their complete protection in such a duty we think medical practitioners would not be at all anxious to undertake the task. Dr. Mercier, in criticising the paper, ridicules the suggestion that the legislature would give the power to any two medical practitioners to deprive a man of his liberty for a term of six months for drunkenness. Probably this is quite true, but it is well to emphasise the position that few medical practitioners desire any such power. Indeed, there are medical practitioners who would be glad to be relieved of the responsibility involved in an ordinary lunacy certificate.

### Out-Patient Departments and the Poor Law.

In an elaborate article on the "Legal Poor of London," the *Times* of December 26 draws attention to the difficulty created in the distribution of parochial medical relief by the ease and readiness with which patients are admitted for treatment in the out-patient departments of the general hospitals. Refusal on adequate grounds is of no avail when the applicant can retort that he can get the treatment he requires without any trouble by applying to any hospital in the neighbourhood. This state of matters, it is contended, renders useless the efforts of the Poor-law authorities to stimulate people to provide for themselves; and, further, the usual hospital arrangements tempt many who could pay for medical relief to become occasional recipients of charity, and, by degrees, habitual paupers. There is another item in the charge, and it is a serious one. The provision of gratuitous medical relief without inquiry, we are told, is a serious obstacle to the promotion of provident institutions. Many refuse to make arrangements to secure for themselves medical attendance in times of sickness by the payment of a small weekly sum, and they do so on the express ground that they can obtain gratuitous treatment at the hospitals. It is such facts, and experiences as these which afford ground for the very serious conclusion that the hospitals "have done more to demoralise the community and to increase the number chargeable to the Poor-law than any other existing institution." This is an aspect of the out-patient department that cannot too frequently and too emphatically be pressed upon public attention. That the general practitioner has his grievance against the hospitals we have always recognised. But his sufferings, we fear, will not stir the sympathy of the community. Such a statement as that endorsed by the *Times*, however, presents the existing out-patient practice as an important public question which demands the attention of all interested in social problems.

### Pharmacology and Therapeutics of the Formates.

The experimental pharmacologist occasionally produces and publishes conclusions which are distinctly startling to the practising physician; the laboratory result and clinical experience are brought into acute contradiction, and the precise measurements which accompany the former seem to establish a strong presumption in its favour. One of the latest remedies to suffer from this divergence of view is the group of the formates. Much has been written of recent years of the value of these salts as tonics for the nervous and muscular apparatus, and the voice of the advertiser with its usual strident tones has been heard on the subject. Yet allowing for the exaggeration which seems to be the inevitable accompaniment of commercial, and sometimes, it must be allowed, also of professional activity, it can hardly be doubted that the formates have some degree of therapeutic value. Now, however, appears a pharmacological and experimental research in which these salts are, according to laboratory tests, shown to be useless or even harmful. All of them, we are told, can be shown to accelerate rather than to retard fatigue, and each member of the group exerts, it is said, a toxic action on the circulatory and muscular systems. This depressant effect is particularly marked in the case of potassium formate, the metallic ion in which has a depressant influence of its own. Even sodium formate, however, does not escape condemnation, and the announcement is made "that it might impair the strength of a debilitated patient," while calcium formate receives "faint praise" in the sentence, "it might be a serviceable form in which to exhibit lime." Such briefly is the story recently told to the Edinburgh Medico-Chirurgical Society, by Dr. A. Goodall and Miss Isabel Mitchell, B.Sc. The other aspect of the matter was presented by Dr. Geo. A. Gibson, who testified to the value of formic acid on the basis of his own clinical experience; in chorea, in particular, he had found it most valuable as an agent for steadying the muscular system. Similarly, Dr. Claude B. Ker said that the toxic effects described by the authors of the paper were not met with, and that the value of the formates in cardiac failure and in diphtheritic and other forms of paralysis was beyond question.

